# Implications of Medicare Negotiation and Most-Favored-Nation Pricing for Cancer Medicine Costs

**DOI:** 10.1001/jamahealthforum.2026.0509

**Published:** 2026-05-01

**Authors:** Thomas J. Hwang, Ariadna Tibau, Aaron S. Kesselheim, Kerstin Noelle Vokinger

**Affiliations:** 1 Cancer Innovation and Regulation Initiative, Division of Pharmacoepidemiology and Pharmacoeconomics, Brigham and Women’s Hospital and Harvard Medical School, Boston, Massachusetts; 2 Academic Chair for Regulation in Law, Medicine, and Technology, Department of Health Sciences and Technology, ETH Zurich, Zurich, Switzerland; 3 Program On Regulation, Therapeutics, And Law (PORTAL), Division of Pharmacoepidemiology and Pharmacoeconomics, Brigham and Women’s Hospital and Harvard Medical School, Boston, Massachusetts; 4 Academic Chair for Regulation in Law, Medicine, and Technology, Faculty of Law, University of Zurich, Zurich, Switzerland

## Abstract

This cross-sectional study explores expenditures, negotiated pricing, and potential additional savings for top-selling cancer products in Medicare Part B and Part D.

## Introduction

In December 2025, the Centers for Medicare & Medicaid Services (CMS) proposed models for most-favored-nation (MFN) pricing for brand-name pharmaceuticals in Medicare Part B^1^ and Part D.^2^ In January 2026, CMS also announced the start of the third cycle of Medicare drug price negotiations authorized by the Inflation Reduction Act. The first 2 cycles of negotiations, covering 25 high-cost products, were estimated by Medicare to save, on average, 22% and 44% of net drug costs in 2023 and 2024, respectively.^3,4^

Cancer medicines are costly for beneficiaries and Medicare, with limited rebates and discounts.^5^ For top-selling cancer products, we estimated potential savings under both MFN pricing (linked to pricing in comparable countries) and Medicare negotiation.

## Methods

Total Medicare Part B (physician-administered products) and Part D (outpatient retail drugs) spending data in 2024 for brand-name cancer drugs were obtained from Medicare Drug Spending databases. Following CMS’ proposal,^1^ we focused on drugs with more than $100 million annual Medicare spending and excluded generic and biosimilar products. In accordance with 45 CFR §46.104, this cross-sectional study was exempt from institutional review board review because publicly available and aggregated data were used. We followed the STROBE reporting guideline.

We obtained reported list price discounts from the 2 prior cycles of Medicare negotiation.^3^ For each top-selling cancer drug, we compared reported prices for included products (wholesale acquisition costs for Part D drugs and average sales prices for Part B drugs) against ex-factory unit prices in a basket of 19 comparator countries (with gross domestic product per capita at least 60% that of the US) from the CMS Proposed Rules (eTables 1 and 2 in Supplement 1).^1,2^ We considered 2 cohorts: cancer drugs already selected for negotiation and all top-selling cancer drugs. Reductions in Medicare spending in 2024 under MFN pricing (either lowest or average international price) were then estimated for both cohorts (eMethods in Supplement 1). We also estimated additional reductions in Medicare spending from MFN pricing, beyond the discounts from the first 2 negotiation cycles. Data analysis was performed with Stata, version 16 (StataCorp LLC).

## Results

In 2024, total Medicare spending on included brand-name cancer drugs was $49.6 billion ($17.6 billion in Part B; $32.0 billion in Part D) for 76 products. Nine cancer drugs were selected for Medicare negotiation (Imbruvica in cycle 1; Xtandi, Ibrance, Pomalyst, and Calquence in cycle 2; and Verzenio, Erleada, Lenvima, and Kisqali in cycle 3), representing $15.8 billion in annual Medicare spending (Table).

**Table.** Comparison of Reported List Price Discounts Under Medicare Negotiation and MFN Pricing for Selected Cancer Drugs^a^

Drug name (US sponsor)	Year of first FDA approval	2024 Total Medicare spending, million, $	Medicare Part B vs Part D	Reported negotiated discount, %	Estimated discount, %^b^	
					Under average MFN pricing	Under lowest MFN pricing
**Selected for negotiation cycles 1 and 2, 2024-2025**						
Imbruvica (AbbVie and Johnson & Johnson)	2013	2371.9^c^	Part D	38	70	85
Xtandi (Astellas and Pfizer)	2012	3401.1	Part D	48	80	93
Ibrance (Pfizer)	2015	2036.2	Part D	50	85	91
Pomalyst (Bristol Myers Squibb)	2013	2150.6	Part D	60	73	93
Calquence (AstraZeneca)	2017	1703.1	Part D	40	63	71
**Selected for negotiation cycle 3, 2026 and ongoing**						
Verzenio (Eli Lilly and Company)	2017	1056.8	Part D	Ongoing	78	86
Erleada (Johnson & Johnson)	2018	1386.3	Part D	Ongoing	80	91
Lenvima (Eisai)	2015	875.6	Part D	Ongoing	94	98
Kisqali (Novartis)	2017	798.4	Part D	Ongoing	85	93

Abbreviations: FDA, US Food and Drug Administration; MFN, most-favored-nation.

^a^ The table includes cancer drugs selected for Medicare negotiation as of January 2026. Medicare Part B primarily covers physician-administered products, such as infused, injectable, and inhaled drugs, and Part D covers outpatient retail drugs.

^b^ Estimated discount used average international price in included comparator countries (average MFN pricing) or lowest available international comparator price (lowest MFN pricing). The eMethods in Supplement 1 provides further information.

^c^ Data, obtained from Medicare negotiation,^3^ reflect the reported total Medicare expenditures in 2023 for ibrutinib (Imbruvica), given the drug’s selection for negotiation in 2024.

The median (IQR) ratios of MFN pricing to US unit prices ranged from 0.19 (0.12-0.28) using the lowest international price to 0.35 (0.23-0.45) using the average international price. For the 5 cancer drugs in cycles 1 and 2 with negotiated prices, the weighted average discount from list prices was 47.4%. Applying MFN pricing would have reduced Medicare spending in 2024 by an additional $3.2 to $4.7 billion (spending reductions of 27.8%-40.5%). Applying MFN pricing to all included cancer drugs would have reduced annual Medicare spending in 2024 by $27.1 to $35.4 billion (Figure).

**Figure.**
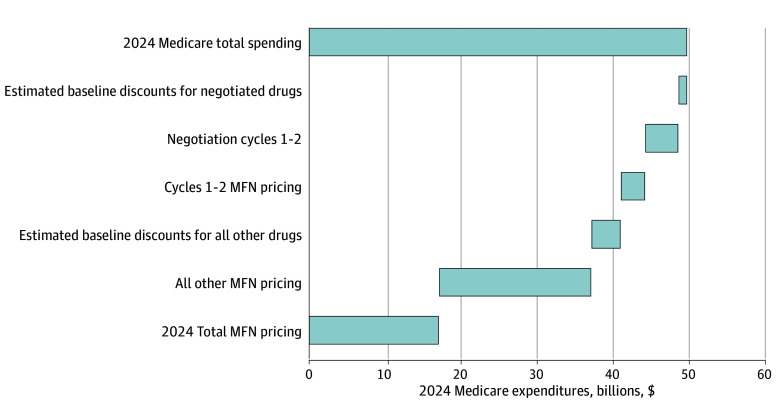
Waterfall Plot of Estimated Reduction in 2024 Medicare Expenditures on Cancer Drugs With Medicare Negotiation and Most-Favored-Nation (MFN) Pricing Total gross Medicare expenditures on included cancer drugs in 2024 were $49.6 billion. Figure shows estimated baseline discounts for negotiated drugs (eMethods in Supplement 1), estimated reductions in spending from Medicare negotiation (reported discounts from negotiation cycles 1 and 2), and incremental possible reductions in spending from application of MFN pricing using the average international price from the basket of comparator countries specified by the Centers for Medicare & Medicaid Services in its Proposed Rules.

## Discussion

Savings from Medicare negotiation and MFN pricing may be greater for cancer drugs than other drug classes because of low average rebates currently for cancer drugs^6^ and because international prices were 65% to 81% lower than US prices. Applying MFN pricing (as CMS proposed for Part B and Part D) could have substantially reduced Medicare cancer drug spending in 2024 beyond the negotiated prices achieved thus far. However, the long-term sustainability of these savings is uncertain, as such a policy would be prone to manufacturer gaming. For example, manufacturers may delay market entry in small countries to prevent their lower prices from being referenced and may transition to confidential discounts and managed-entry agreements. To counteract these possible responses, CMS could remove smaller countries from the reference basket and consider limiting MFN pricing to drugs associated with low clinical benefit.

Study limitations include our focus on high-cost cancer drugs. Additionally, results may not be generalizable to cancer drugs with low Medicare spending.

Given their high costs, cancer drugs are likely to be frequently selected for Medicare price negotiation. Policymakers should explore sharing more of the substantial potential savings from MFN pricing with patients by eliminating out-of-pocket costs for highly effective therapies.

## Notes

Supplement 1.
eMethods.
**eTable 1.** Basket of Comparator Countries **eTable 2.** List of Included Products in Study Cohort

Supplement 2.
Data Sharing Statement
