# Supplementary material for: Implications of Medicare Negotiation and Most-Favored-Nation Pricing for Cancer Medicine Costs
Source: JAMA Health Forum. 2026 May 1;7(5):e260509. doi: 10.1001/jamahealthforum.2026.0509 (PMC13135206; doi:10.1001/jamahealthforum.2026.0509)
Supplement: Supplement 1. — eMethods. eTable 1. Basket of Comparator Countries eTable 2. List of Included Products in Study Cohort [file jamahealthforum-e260509-s001.pdf]

Supplemental Online Content

Hwang TJ, Tibau A, Kesselheim AS, Vokinger KN. Implications of Medicare negotiation and most-favored-nation pricing for cancer medicine costs. *JAMA Health Forum*. 2026;7(5):e260509. doi:10.1001/jamahealthforum.2026.0509

eMethods

eTable 1. Basket of Comparator Countries

eTable 2. List of Included Products in Study Cohort

This supplementary material has been provided by the authors to give readers additional details about their work.

© 2026 Hwang TJ et al. *JAMA Health Forum*

eMethods.

Cohort Inclusion

We identified brand-name cancer drugs with total annual Medicare spending of at least $100 million across Medicare Parts B and D using data from the Medicare Drug Spending Dashboard for 2024 (most recent year available). For each included top-selling cancer drug, we identified Part D vs. Part B status (Part B drugs eligible for negotiation beginning in 2026) and generic or biosimilar availability. We excluded generic and biosimilar drugs as well as brand-name drugs already subject to generic or biosimilar competition as of January 1, 2026.

Negotiation Discounts

We obtained the list of selected products in the first (ibrutinib) and second (acalabrutinib, enzalutamide, palbociclib, and pomalidomide) cycles of Medicare negotiation, as well as their reported discounts from list price, from Medicare. Weighted average discounts from Medicare negotiation were calculated using total gross expenditures published by Medicare in the year of negotiation announcement.

International Prices

We obtained wholesale acquisition costs (for Part D drugs) and average sales prices (for Part B drugs) for all included products from Micromedex (Red Book) and Medicare, respectively, as of May 20, 2025. The basket of comparator countries (60% of US Gross Domestic Product [GDP] per capita according to the Administration) was obtained from the Centers for Medicare and Medicaid Services' Proposed Rules on most-favored nation pricing (see reference list). Publicly available ex-factory unit prices were obtained for all proposed benchmark countries (19 countries meeting the Proposed Rule's criteria) using official price registers (France, Germany, Switzerland, and United Kingdom) and supplemented with data from a commercially available drug pricing database (NAVLIN Eversana), which is cited in the Proposed Rules as a source of international pharmaceutical pricing data. Pricing data were manually validated by consensus by two reviewers for a 5% sample (TJH and AT). Median international-to-US price ratios were calculated using the lowest available price and the average price in the basket of comparator countries.

Estimated Possible Savings

We considered two subgroups of cancer drugs: (1) those already selected (1 in first cycle, 4 in second cycle of negotiations) for Medicare negotiation with announced discounts as of January 2026 and (2) all included cancer drugs. We assumed current Medicare net prices using the class-average rebate level per the Medicare Payment Advisory Commission and, for each subgroup, we estimated the reductions in Medicare net spending with Medicare negotiation alone (using reported Medicare discounts from list price in the first two cycles of negotiation) and applying "most-favored-nation" pricing (with the average international price in the basket of comparator countries) for the second cohort of all included drugs. The waterfall plot was constructed with the following components: (i) estimated baseline (pre-negotiation) discounts using the class-average rebate level; (ii) incremental reduction in Medicare expenditures from reported Medicare negotiation from list price; (iii) incremental reduction in Medicare expenditures from applying most-favored nation pricing to first subgroup of cancer drugs selected for negotiation in first two cycles; (iv) estimated baseline discounts for all non-selected cancer drugs using class-average rebate level; and (v) incremental reduction in Medicare expenditures from applying most-favored nation pricing to the second subgroup of all other (non-selected) cancer drugs.

© 2026 Hwang TJ et al. *JAMA Health Forum*

eTable 1. Basket of Comparator Countries

	Reference Basket
Australia	●
Austria	●
Belgium	●
Canada	●
Czechia	●
Denmark	●
France	●
Germany	●
Ireland	●
Israel	●
Italy	●
Japan	●
Netherlands	●
Norway	●
Sweden	●
Switzerland	●
South Korea	●
Spain	●
United Kingdom	●

Notes: The basket of comparator countries (60% of US Gross Domestic Product [GDP] per capita) was defined by the Centers for Medicare and Medicaid Services in the Proposed Rules using latest US Central Intelligence Agency data on GDP per capita.

eTable 2. List of Included Products in Study Cohort

Generic Name	Brand Name	Negotiation
ibrutinib	Imbruvica	Cycle 1
acalabrutinib	Calquence	Cycle 2
enzalutamide	Xtandi	Cycle 2
palbociclib	Ibrance	Cycle 2
pomalidomide	Pomalyst	Cycle 2
abemaciclib	Verzenio	
ado-trastuzumab emtansine	Kadcyla	
alectinib	Alecensa	
alpelisib	Piqray	
apalutamide	Erleada	
asciminib	Scemblix	
atezolizumab	Tecentriq	
avapritinib	Ayvakit	
avelumab	Bavencio	
axitinib	Inlyta	
belzutifan	Welireg	
bosutinib	Bosulif	
brentuximab	Adcetris	
cabazitaxel	Jevtana	
cabozantinib s-malate	Cabometyx	
capivasertib	Truqap	
capmatinib	Tabrecta	
carfilzomib	Kyprolis	
cemiplimab	Libtayo	
cetuximab	Erbitux	
dabrafenib	Tafinlar	
daratumumab	Darzalex	
daratumumab hyaluronidase	Darzalex Faspro	
darolutamide	Nubeqa	
dostarlimab	Jemperli	
durvalumab	Imfinzi	
elacestrant	Orserdu	
elotuzumab	Empliciti	
enasidenib	Idhifa	
encorafenib	Braftovi	
enfortumab	Padcev	
fam-trastuzumab	Enhertu	
fruquintinib	Fruzaqla	
gilteritinib	Xospata	
ipilimumab	Yervoy	
ivosidenib	Tibsovo	
ixazomib	Ninlaro	
lenvatinib	Lenvima	
lorlatinib	Lorbrena	
lurbinectedin	Zepzelca	
momelotinib	Ojjaara	
niraparib	Zejula	
nivolumab	Jakafi	
nivolumab	Opdivo	
nivolumab-relatlimab	Opdualag	
obinutuzumab	Gazyva	
ofatumumab	Kesimpta	
olaparib	Lynparza	
osimertinib	Tagrisso	
pacritinib	Vonjo	
panitumumab	Vectibix	
pembrolizumab	Keytruda	
pertuzumab	Perjeta	
pertuzumab-trastuzumab	Phesgo	
pirtobrutinib	Jaypirca	
polatuzumab vedotin	Polivy	
ponatinib	Iclusig	
ramucirumab	Cyramza	
regorafenib	Stivarga	
relugolix	Orgovyx	
ribociclib	Kisqali	
sacituzumab govitecan	Trodelvy	
sotorasib	Lumakras	
teclistamab	Tecvayli	
tivozanib	Fotivda	
trametinib	Mekinist	
trifluridine/tipiracil	Lonsurf	
tucatinib	Tukysa	
venetoclax	Venclexta	
vismodegib	Erivedge	
zanubrutinib	Brukinsa	

© 2026 Hwang TJ et al. *JAMA Health Forum*
