# Supplementary material for: Implications of Medicare Negotiation and Most-Favored-Nation Pricing for Cancer Medicine Costs
Source: JAMA Health Forum. 2026 May 1;7(5):e260509. doi: 10.1001/jamahealthforum.2026.0509 (PMC13135206; doi:10.1001/jamahealthforum.2026.0509)
Supplement: Supplement 2. — Data Sharing Statement [file jamahealthforum-e260509-s002.pdf]

Data Sharing Statement

Hwang. Implications of Medicare Negotiation and Most-Favored-Nation Pricing for Cancer Medicine Costs. *JAMA Health Forum*. Published May 01, 2026.
doi:10.1001/jamahealthforum.2026.0509

Data
Data available: Yes
Data types: Data (not involving human participants)
How to access data: Publicly available
When available: With publication

Supporting Documents
Document types: None

Additional Information
Who can access the data: Anyone requesting the data
Types of analyses: Any purpose
Mechanisms of data availability: Publicly available
